# Impact of the COVID-19 pandemic on ophthalmologic outpatient care: experience from an Italian Tertiary Medical Center

**DOI:** 10.1080/07853890.2021.1964036

**Published:** 2021-08-16

**Authors:** Alfonso Savastano, Matteo Ripa, Maria Cristina Savastano, Raphael Kilian, Giorgio Marchini, Stanislao Rizzo

**Affiliations:** a Ophthalmology Unit, “Fondazione Policlinico Universitario A. Gemelli IRCCS”, Rome, Italy; b Catholic University “Sacro Cuore”, Rome, Italy; c Ophthalmology Unit, University of Verona, Verona, Italy; d Consiglio Nazionale delle Ricerche, Istituto di Neuroscienze, Pisa, Italy

**Keywords:** Booked appointments, COVID-19 pandemic, national lockdown, ophthalmic care, performed outpatient visits, personalized medicine

## Abstract

**Purpose:**

To evaluate the impact the COVID-19-related national lockdown has had on Ophthalmologic Outpatient Care in an Italian Tertiary Medical Centre.

**Methods:**

We reviewed all the performances that were carried out as outpatient services at our department between 1 January 2020 and 30 November 2020. We compared data among four different periods: from 1 January 2020 to 17 March 2020 (“Pre-Lockdown”); from 18 March 2020 to 17 May 2020 (Lockdown); from 18 May 2020 to 2 November 2020 (Post-Lockdown) and from 3 November 2020 to 30 November 2020 (Regional Lockdown).

**Results:**

The overall number of performed routine outpatient visits per day (ROVs) was 11,871 (Mean ± SD = 35.76 ± 17.81), whereas booked appointments (BAs) were 21272 (Mean ± SD = 63.86 ± 9.27), meaning a decline in the number of ROVs by 44.01%. (Mean ± SD = 28.10 ± 12.11, *p*<.001). Post-Lockdown and Regional Lockdown clinical activities, dropped respectively by 31 and 25.14% (38.87 ± 3.88 vs. 56.34 ± 11.06, *p*<.001 and 6.04 ± 4.51 vs. 56.34 ± 11.06 *p*<.001). The number of BAs per day decreased during the pandemic, going from a mean of 77.81 ± 2.57 booked appointments per day before the lockdown, to a mean of 53.14 ± 4.94, 61.80 ± 4.62 and 72.07 ± 1.09 appointments per day respectively during the lockdown, the post-lockdown and the regional lockdown periods.

**Conclusions:**

During the various lockdown periods, at our institution the volume of outpatient ophthalmological visits drastically dropped. This testifies the dramatic impact the COVID-19 pandemic has had on the supply of ophthalmic care.

## Introduction

It has been more than a year since the Severe acute respiratory syndrome Coronavirus 2 (Sars-CoV-2) infection first started disseminating worldwide . In order to mitigate the unprecedented massive spread of the virus, on 9 March 2020, the Italian Government imposed a nation-wide Lockdown that included measures like the ban on mass gatherings and events, as well as the ban on any kind of meetings except for urgent reasons. In light of this overall scenario, to reduce the chance of viral transmission among patients and healthcare personnel, providers started deferring routine outpatient department visits [1]. As the number of cases went down, after 69 days of lockdown, in May 2020 Italy started “Phase 2” of the national emergency program by undergoing a gradual relaxation of the Lockdown measures. After a short period of relative improvement in the diffusion of the virus, from September 2020 onwards, the incidence of infection restarted growing, up to the moment, on October 14, when the number of new cases per day exceeded those we had during the highest peak in March. It was November 4, 2020, when a new Lockdown was announced by the government. This time, the country’s regions were divided into three different zones (i.e. red, orange and yellow zones). Depending on the severity of viral diffusion, the regions with the darkest colour (i.e. red) were classified as having the highest risk, while those with the lightest colour (i.e. yellow) as having the lowest risk of contagion. Orange regions stood right in the middle. The red zones were put under strict lockdown regulations, similar to those we knew from March to May 2020. A less strict lockdown was applied in the orange zones, while the “yellow regions” underwent only few restrictions.

Healthcare systems have been severely upset all over the world. In fact, the Sars-Cov-2 pandemic has redirected lots of the energies, time and financial resources that were usually invested into ordinary care, to the fight against the virus. Despite the presence of many studies reporting on the systemic and also ocular manifestations of Sars-Cov-2 infected patients [2,3], analyses of the impact that the virus has had on Ophthalmic Healthcare are still lacking. The latter would be useful at providing a forecast of the future adversities ophthalmologists are going to face. The aim of this study was to evaluate the effect the COVID-19 pandemic and the related national containment measures have had on the Ophthalmologic Outpatient Care at the Ophthalmology Department of the “A. Gemelli Polyclinic University Foundation IRCSS” between 1 January 2020 and 30 November 2020.

## Methods

This retrospective cross-sectional study was conducted in accordance with the ethical standards of the institutional research committee and with the tenets of the Declaration of Helsinki. The study protocol was approved by the Catholic University/Fondazione Policlinico Gemelli IRCCS Institutional Ethical Committee (protocol ID number: 3680/20).

We performed a retrospective review of the electronic medical records of all patients that presented to the Ophthalmology Department between 1 January 2020, and 30 November 2020. These data were compared among four periods, i.e. 1 January 2020 – 8 March 2020 (Pre-Lockdown Period), 9 March 2020 – 18 May 2020 (Lockdown Period), 19 May 2020 – 2 November 2020 (Post-Lockdown Period) and 3 November 2020 – 30 November 2020 (Regional Lockdown Period).

Assessed data included reports of routine outpatient visits (ROVs) and diagnostic exams (i.e. Electroretinography [ERG], visual evoked potential [VEP], Ocular Echography, Fluorescein angiography [FA] and optical coherence tomography [OCT]), which accounted for “Clinical Activity “(CA), but also of routine outpatient treatments (i.e. Intravitreal injections, retinal Laser, YAG Laser, cross-linking and other outpatient surgeries). All of the above-mentioned performances were conducted in our Ophthalmology Outpatient Departments (i.e. Glaucoma, Cornea and Refractive Surgery, Medical Retina, Paediatric Retina, Ocular Oncology, Orbital Lacrimal and Ophthalmic Plastic Surgery, Electrophysiology, Uveitis and Ocular Inflammation, Paediatric Ophthalmology). In order to simplify calculations, different activities for the same patient, on the same day, were considered independently from one another.

Statistical calculations were performed using Statistical Package for Social Sciences (version 27.0, SPSS Inc., Chicago, IL, USA). To detect differences from normal distribution, a Shapiro–Wilk’s test was performed for all variables. Means and standard deviation (SD) were computed for all quantitative variables. Continuous variables were compared by conducting a Student’s *t* test for independent variables. Statistical significance of the differences between groups for qualitative variables was assessed using Fisher’s exact test. Pairwise comparisons were performed using Dunn’s procedure with Bonferroni correction for multiple comparisons. Multivariate analysis of variance (MANOVA) was performed to evaluate whether there were any statistically significant differences among the means of the independent groups. A Post Tukey Analysis was performed. A *p* value <.05 was determined to be statistically significant.

## Results

Despite the overall number of BAs being 21272 (Mean ± SD per day: 63.86 ± 9.27), between January 1 and November the 30 the total number of performed ROVs was only 11871 (Mean ± SD per day: 35.76 ± 17.81), meaning a decline by 44.01% (28.10 ± 12.11).

**Figure 1.**
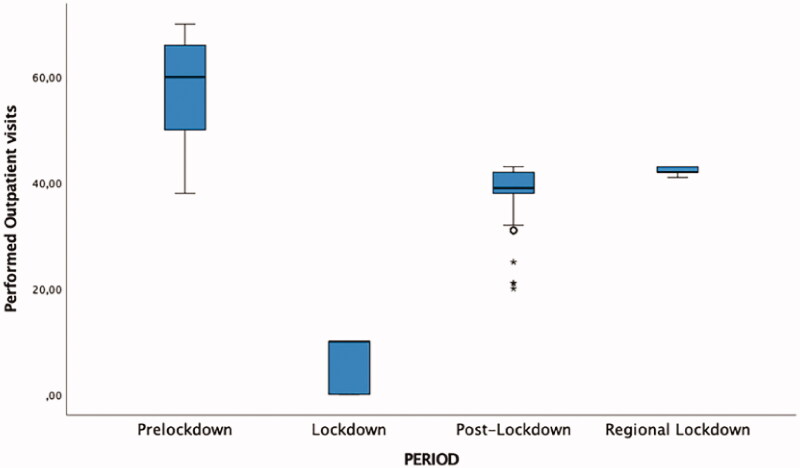
Performed outpatients visits correlated to the various pandemic lockdown periods. The values were significantly different between groups. *p*-Values were presented: Lockdown period versus Pre-Lockdown period, *p* = .001; Post-Lockdown versus Pre-Lockdown period *p* = .001 and Regional Lockdown vs. Pre-Lockdown period *p* = .001. Tukey *post hoc* analysis and Student–Newman–Keuls *post hoc* analysis revealed that the difference between Pre-Lockdown performed visits and Lockdown performed visit was statistically significant (*p* = .001; 95%CI = 47.63; 52.97).

**Figure 2.**
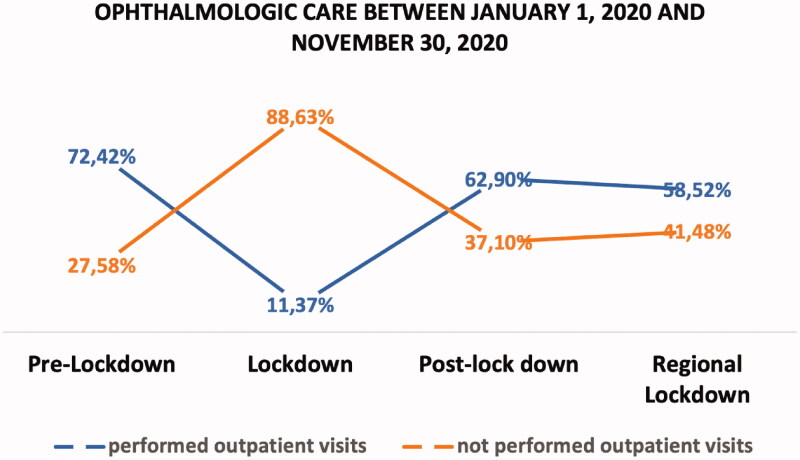
Graph shows rate of performed and not performed outpatient visits throughout the COVID-19 Pandemic.

The distribution of performed outpatient visits and the comparison between performed and not-performed outpatient visits during the different time intervals is shown in Figures 1 and 2 respectively.

During the Pre-Lockdown Period (1 January 2020–8 March 2020) ROVs declined by 27.58% compared to the booked appointments per day (i.e. 77.81 ± 2.57). Specifically, the number of not-performed visits during the Pre-lockdown period was 21.46 ± 10.83 visits per day, *p* < .001. During the Lockdown Period (9 March 2020–18 May 2020) numbers went down by up to 88.64% (47.10 ± 6.17 visits per day, *p* < .001). During the Post-Lockdown and the Regional Lockdown phases, on the other hand, ROVs declined by 37.10% (Mean ± SD: 22.93 ± 6.31 visits per day, *p* < .001) and by 41.48% (Mean ± SD: 29.89 ± 1.31 visits per day, *p* < .001), respectively.

Overall, lockdown CA declined by 89.27% (6.04 ± 4.51 visits per day vs. 56.34 ± 11.06 visits per day, *p* = .0015) when compared to the Pre-Lockdown period, whereas Post-Lockdown and Regional Lockdown CA declined respectively by 31% and 25.14% (38.87 ± 3.88 visits per day vs. 56.34 ± 11.06 visits per day, *p* < .001 and 6.04 ± 4.51 visits per day vs. 42.18 ± 0.61 visits per day, *p* < .001). Booked appointments per day on the other hand, decreased from a mean of 77.81 (SD ± 2.57) during Pre-Lockdown to 53.14 ± 4.94, 61.80 ± 4.62 and 72.07 ± 1.09, respectively during Lockdown, Post-Lockdown and Regional Lockdown periods.

**Figure 3.**
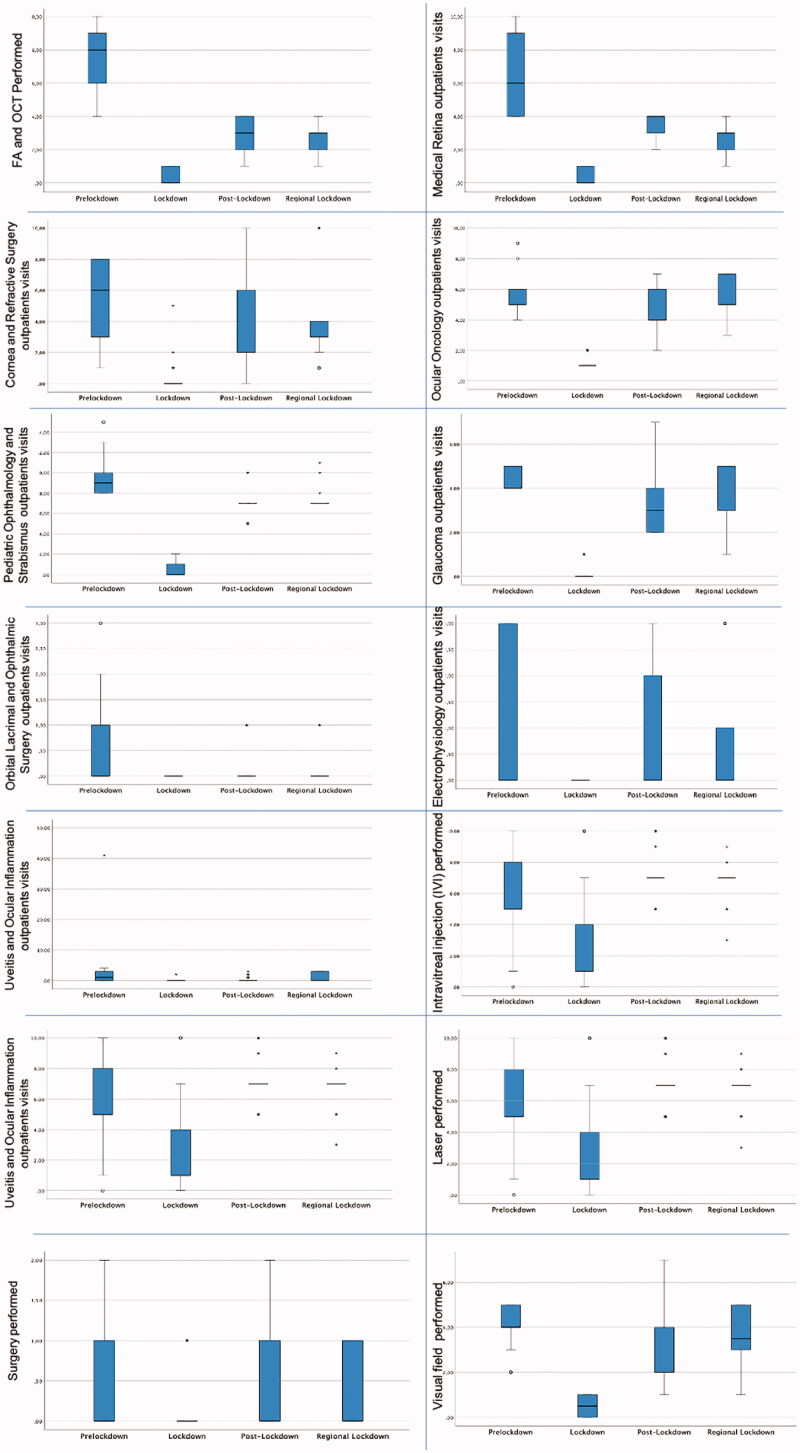
Performed outpatients visits, exams and treatments during the COVID-19 pandemic. The values were significantly between groups, *p*-values was presented: Lockdown period versus Pre-Lockdown period, *p* = .001; Post-Lockdown versus Pre-Lockdown period *p* = .001 and regional Lockdown vs. Pre-lockdown period *p* = .001.

As follows, we report the data that were obtained among the different activities performed at our department. Results are displayed as percentages of decrease among various periods and as the difference between the mean number of visits per day during any of the lockdown periods and the number of visits per day during the Pre-Lockdown Period. Exact distribution of performances during the different time periods is shown in Figure 3 and Table 1.

**Table 1.** Outpatient department clinical activity during the Covid-19 pandemic.

	Pre-lockdown	Lockdown	Post-lockdown	Regional lockdown
Medical retina (visits/day)				
Booked	8.85 ± 1.79	6.24 ± 0.67	5.74 ± 0.92	4.54 ± 0.64
Performed	6.40 ± 2	0.44 ± 0.50	3.63 ± 0.58	2.64 ± 0.68
Not performed	2.45 ± 1.66	5.80 ± 0.73	2.10 ± 0.96	1.89 ± 1.10
FA and OCT (visits/day)				
Booked	10.15 ± 0.93	6.44 ± 0.85	4.43 ± 1.23	4.86 ± 0.52
Performed	7.52 ± 2.11	0.47 ± 0.50	2.71 ± 1.09	2.57 ± 0.69
Not performed	2.63 ± 1.87	5.97 ± 0.78	1.71 ± 1.27	2.29 ± 0.85
Cornea and refractive surgery (visits/day)				
Booked	7.12 ± 2.55	4.53 ± 3.34	4.82 ± 2.98	7.07 ± 2.76
Performed	5.15 ± 2.46	0.26 ± 0.72	3.34 ± 2.30	4.21 ± 2.88
Not performed	1.97 ± 1.71	4.27 ± 3.44	1.49 ± 1.80	2.86 ± 2.37
Paediatric ophthalmology and strabismus (visits/day)				
Booked	13.55 ± 0.99	10.33 ± 0.74	13.83 ± 2.74	15.89 ± 1.10
Performed	9.21 ± 1.54	0.33 ± 0.50	6.68 ± 0.85	7.29 ± 0.94
Not performed	4.34 ± 1.75	10.00 ± 0.78	7.15 ± 3.08	8.61 ± 1.20
Ocular oncology (visits/day)				
Booked	7.00 ± 1.15	4.54 ± 1	6.96 ± 1.40	9.79 ± 0.99
Performed	5.43 ± 1.09	1.21 ± 0.41	4.53 ± 1.52	5.57 ± 1.20
Not performed	1.57 ± 1.22	3.33 ± 1.05	2.43 ± 1.77	4.21 ± 1.37
Glaucoma (visits/day)				
Booked	6.28 ± 0.45	5.56 ± 0.67	5.34 ± 1.99	5.79 ± 0.42
Performed	4.10 ± 1.02	0.50 ± 0.50	3.15 ± 1.50	3.64 ± 1.25
Not performed	1.42 ± 0.86	6.39 ± 1.11	2.14 ± 1.51	1.71 ± 1.1
Visual fields (visits/day)				
Booked	5.52 ± 0.70	6.89 ± 1.00	5.29 ± 1.96	5.36 ± 0.87
Performed	1.82 ± 0.80	1.82 ± 0.80	1.82 ± 0.80	1.82 ± 0.80
Not performed	1.82 ± 0.80	1.82 ± 0.80	1.82 ± 0.80	1.82 ± 0.80
Orbital lacrimal and ophthalmic plastic surgery (visits/day)				
Booked	0.93 ± 0.91	0.66 ± 0.87	0.37 ± 0.71	0.36 ± 0.78
Performed	0.49 ± 0.70	0.00 ± 0.00	0.15 ± 0.36	0.18 ± 0.39
Not performed	0.43 ± 080	0.66 ± 0.87	0.22 ± 0.63	0.18 ± 0.61
Electrophysiology (visits/day)				
Booked	1.88 ± 1.31	1.49 ± 2.09	1.34 ± 1.07	1.25 ± 1.27
Performed	1.03 ± 1.34	0.00 ± 0.00	0.69 ± 0.92	0.57 ± 1.07
Not performed	0.85 ± 1.10	1.49 ± 2.09	0.65 ± 1.10	0.68 ± 1.06
Uveitis and ocular inflammation (visits/day)				
Booked	2.45 ± 1.42	0.57 ± 1.14	0.50 ± 0.81	1.54 ± 1.37
Performed	2.04 ± 5.08	0.03 ± 0.24	0.28 ± 0.55	1.00 ± 1.33
Not performed	0.40 ± 4.99	0.54 ± 1.10	0.22 ± 0.65	0.54 ± 1.04
IVI (visits/day)				
Booked	8.51 ± 1.12	3.49 ± 3.10	8.86 ± 1.82	9.68 ± 2.00
Performed	6.09 ± 2.03	2.21 ± 2.51	7.13 ± 1.17	6.79 ± 1.10
Not performed	2.42 ± 2.00	1.27 ± 2.55	1.73 ± 1.79	2.89 ± 1.64
Retinal and Yag laser (visits/day)				
Booked	8.51 ± 1.12	3.49 ± 3.10	8.86 ± 1.82	9.68 ± 2.00
Performed	6.09 ± 2.03	2.21 ± 2.51	7.13 ± 1.17	6.79 ± 1.10
Not performed	2.42 ± 2.00	1.27 ± 2.55	1.73 ± 1.79	2.89 ± 1.64
Corneal cross-linking (CXL) (visits/day)				
Booked	8.51 ± 1.12	3.49 ± 3.10	8.86 ± 1.82	9.68 ± 2.00
Performed	6.09 ± 2.03	2.21 ± 2.51	7.13 ± 1.17	6.79 ± 1.10
Not performed	2.42 ± 2.00	1.27 ± 2.55	1.73 ± 1.79	2.89 ± 1.64
Outpatient surgery (visits/day)				
Booked	0.54 ± 0.61	0.07 ± 0.26	0.50 ± 0.71	0.54 ± 0.51
Performed	0.37 ± 0.55	0.07 ± 0.26	0.42 ± 0.68	0.39 ± 0.50
Not performed	0.16 ± 0.41	0.00 ± 0.00	0.08 ± 0.33	0.14 ± 0.36

Mean and standard deviation (SD) of the different types of performances per day.

### Medical retina service

Compared to the Pre-Lockdown phase, the total number of performed retinal visits per day decreased by 93.08% during the Lockdown Period (0.44 ± 0.50 vs. 6.40 ± 2, *p* < .001), by 43.23% during Post-Lockdown Period (3.63 ± 0.58 vs. 6.40 ± 2, *p* < .001) and by 58.72% during Regional Lockdown Period (2.64 ± 0.68 vs. 6.40 ± 2, *p* < .001).

### Fluorescein angiography (FA) and optical coherence tomography (OCT)

The total number of FA and OCT performed decreased by 93.73% during Lockdown Period (0.47 ± 0.50 vs. 7.52 ± 2.11, *p* < .001), by 63.94% during Post-Lockdown (2.71 ± 1.09 vs. 7.52 ± 2.11, *p* < .001) and by 65.82% during Regional Lockdown Period (2.57 ± 0.69 vs. 7.52 ± 2.11, *p* < .001).

### Corneal service and refractive surgery

Performed visits per day decreased by 95.01% during Lockdown Period (0.26 ± 0.72 vs. 5.15 ± 1.71, *p* < .001), by 35.23% during Post-Lockdown Period (3.34 ± 2.30 vs. 5.15 ± 1.71, *p* < .001) and by 18.16% during Regional Lockdown Period (4.21 ± 2.88 vs 5.15 ± 1.71, *p* < .001).

### Paediatric ophthalmology and strabismus services

Daily outpatient visits decreased by 96.43% during Lockdown Period (0.33 ± 0.50 vs. 9.21 ± 1.54, *p* < .001), by 27.50% during Post-Lockdown Period (6.68 ± 0.85 vs. 9.21 ± 1.54, *p* < .001) and by 20.88% during Regional Lockdown Period (7.29 ± 0.94 vs. 9.21 ± 1.54, *p* < .001).

### Ocular oncology service

The total number of performed visits per day decreased by 77.65% during Lockdown Period (1.21 ± 0.41 vs. 5.43 ± 1.09, *p* < .001) and by 16.56% during Post-Lockdown Period (4.53 ± 1.52 vs. 5.43 ± 1.09, *p* < .001). During Regional Lockdown Period on the other hand, there was a regain of activity by 2.53% (5.57 ± 1.20 vs. 5.43 ± 1.09, *p* < .001).

### Glaucoma service

The number of performed visits decreased by 97.44% during Lockdown Period (0.11 ± 0.32 vs. 4.46 ± 0.50, *p* < .001), by 26.60% during Post-Lockdown Period (3.28 ± 1.54 vs. 4.46 ± 0.50, *p* < .001) and by 21.57% during Regional Lockdown period (3.50 ± 1.23 vs. 4.46 ± 0.50, *p* < .001).

### Visual field (VF)

During the Lockdown Period, performed visual fields decreased by 87.82% (0.50 ± 0.50 vs. 4.10 ± 1.02, *p* < .001), by 23.26% during Post-Lockdown Period (3.15 ± 1.50 vs. 4.10 ± 1.02, *p* < .001) and by 11.25% during Regional Lockdown Period (3.64 ± 1.25 vs. 4.10 ± 1.02, *p* < .001).

### Orbital, lacrimal and ophthalmic plastic services

The total number of performed visits and exams decreased by 100% during the Lockdown Period (0 vs. 0.49 ± 0.70, *p* < .001), by 69.61% during Post-Lockdown Period (0.15 ± 0.36 vs. 0.49 ± 0.70, *p* < .001) and by 63.74% during Regional Lockdown Period (0.18 ± 0.39 vs. 0.49 ± 0.70, *p* < .001).

### Electrophysiology service

Activities decreased by 100% during the Lockdown Period (0 vs. 1.03 ± 1.34, *p* < .001), by 32.55% during Post-Lockdown Period (0.69 ± 0.92 vs. 1.03 ± 1.34, *p* < .001) and by 44.51% during Regional Lockdown Period (0.57 ± 1.07 vs. 1.03 ± 1.34, *p* < .001).

### Uveitis and ocular inflammation services

There was a decrease by 98.60% in the number of visits during Lockdown Period (0.03 ± 0.24 vs. 2.04 ± 5.08, *p* < .001), by 86.24% during Post-Lockdown Period (0.28 ± 0.55 vs. 2.04 ± 5.08, *p* < .001) and by 51.09% during Regional Lockdown Period (1 ± 1.33 vs. 2.04 ± 5.08, *p* < .001).

### Intravitreal injections (IVI)

In contrast to the other services, these had a different trend. While the total number of performed IVIs per day decreased by 63.64% during the Lockdown Period (2.21 ± 2.51 vs. 6.09 ± 2.03, *p* < .001), there was a rebound by 17.02% (4.53 ± 1.52 vs. 6.09 ± 2.03, *p* < .001) and by 11.43% (6.79 ± 1.10 vs. 6.09 ± 2.03, *p* < .001), respectively during the Post-lockdown and the Regional Lockdown Periods.

### Retinal, capsular and iris laser treatments

The total number of performed treatments decreased by 81.20% during Lockdown Period (0.16 ± 0.37 vs. 0.84 ± 0.37, *p* < .001) and by 6.15% during Post-lockdown period (0.78 ± 0.62 vs. 0.84 ± 0.37, *p* < .001). There was a regain of activity by 2.55% during the Regional Lockdown period (0.86 ± 0.36 vs. 0.84 ± 0.37, *p* < .001).

### Corneal Cross-Linking (CXL)

There was a 100% reduction of the performances during the Lockdown Period (0 vs. 0.18 ± 0.39, *p* < .001), a 39.82% reduction during the Post-Lockdown period (0.11 ± 0.45 vs. 0.18 ± 0.39, *p* < .001) and a little rebound by 19.64% during the Regional Lockdown Period (0.21 ± 0.27 vs. 0.18 ± 0.39, *p* < .001).

### Outpatient surgeries

During the Lockdown Period, the total number of performed treatments decreased by 80.86% (0.07 ± 0.26 vs. 0.37 ± 0.55, *p* < .001) followed by a rebound by 12.34% during Post-Lockdown Period (0.42 ± 0.68 vs. 0.37 ± 0.55, *p* < .001) and by 5.29% during the Regional Lockdown Period (0.39 ± 0.50 vs. 0.37 ± 0.55, *p* < .001).

## Discussion

The vast majority of the previous studies regarding the role of COVID-19 in the ophthalmologic field, have mainly focussed on pinpointing the incidence and the characteristics of the ocular viral manifestations and on the generation of safety guidelines for ophthalmic practitioners [4]. Herein we reported the impact that the COVID 19 Pandemic and the related national lockdown measures have had on Ophthalmic Care.

After analysing all the medical records registered during this dramatic period, we noted an impressive fall in the total number of ophthalmological outpatient performances.

Every subspecialty at our department had a significant reduction in the number of their activities. This was especially true for the Medical Retina service, in particular with regards to the number of performed retinal diagnostic imaging studies (i.e. FA and OCT). The reduction of the latter led to a forced loss of chronically ill patients’ follow-ups. The fact that the number of intravitreal injections did actually increase during the Post-Lockdown Period, testifies the damage that the lack of treatments and follow-up visits has caused to these patients. People who did not attend the routine visits they actually needed, had to undergo a more aggressive treatment afterwards. Moreover, ocular pathologies necessitating frequent controls (e.g. diabetic retinopathy, retinal occlusive vascular disorders and age-related macular degeneration) tend to be more frequent in elderly fragile patients. Thus, it seems like the latter category of patients suffered and will eventually suffer the most from a general lack of Ophthalmic Care.

In this study we did not actually measure the direct clinical consequences the lockdown measures have had on our patients. However, some hints to what the delay in treatments and in follow-up visits might have caused on the patients’ retinal diseases, are given by some episodes we witnessed in our clinical practice during the pandemic. Particularly, during the lockdown period, a total of 3 patients presented to the ophthalmic emergency room due to complete loss of vision due to a subretinal haemorrhage. It turned out these patients had a known wet age-related-macular-degeneration (AMD) and were all scheduled to receive an anti-VEGF injection during the previous weeks, which they missed due to the “fear” of coming to the hospital.

We can easily imagine how the same kind of events might have taken place, but went unnoticed, also in patients with glaucoma, “the silent thief of sight” (i.e. with an increase in the damage to their visual fields).

Despite the lockdown measures letting people move between different Italian regions and cities for health-related reasons, many of the programmed outpatient visits were deferred. On one hand, this was due to the fear people had of getting the infection in hospitals, which led many of them to cancel their visits, on the other, Ophthalmology departments all around Italy were forced to reduce their activities in order to invest more personnel, time and spaces in the fight against COVID-19 and in order to improve social distancing. Furthermore, we also have to consider that many of our colleagues were unable to actually come to work, either because they got the infection themselves or because they had to precautionary undergo isolation protocols due to a positive contact.

The drastic measures that were introduced by the Italian Government actually finally translated into a slow reduction of the number of fatalities. The visual prognosis of many patients, however, was strongly impaired by the worsening condition of the general Ophthalmologic Outpatient Care. The increased number of outpatient visits in some of the above-mentioned services during the Post-Lockdown and Regional Lockdown Periods (i.e. ocular oncology, laser treatments, CXL and outpatient surgeries) further proves the lack of Ophthalmologic Care there had been during the previous phases.

In order to prevent the infection of patients and personnel in our ophthalmology practices, we adopted new safety measures. Amongst social distancing measures in the waiting rooms and increased frequency of rooms cleaning, we staggered the appointments and made sure the patients who actually had their appointments scheduled, didn’t have any suspicious symptoms the days before. The latter was done by directly calling the patients the day before arrival. Moreover, a triage station had been set up at the ward entrance. Here, patients undergo a touchless body temperature measurement, they are asked to fill-up a questionnaire asking for respiratory symptoms or close contact with infected patients and are finally asked to rub their hands with an alcohol-based solution. Furthermore, consultation-rooms were equipped with custom-made slit lamp breath shields on each slit lamp in order to block the overspray from sneeze [4]. All together, these measures allowed few patients to safely get the ophthalmological management they needed.

Today more than ever, telemedicine has been and should be leveraged to overcome some of the difficulties we as ophthalmologists are currently facing. A simple example of how telemedicine could help to guarantee a good Ophthalmologic Care is the assessment of glaucoma medication tolerance we can do by simply calling the patients. Another, more advanced, very useful application of telemedicine, is the screening of retinal diseases such as vascular retinal diseases, age related maculopathy or diabetic retinopathies [5].

Despite the improvements telemedicine has had in recent years, we understand how important an “Ophthalmological Essential Care service” actually is in order to guarantee many patients’ vision. This is especially true for patients whose pathologies usually necessitate strict follow ups such as glaucoma, retinal diseases and ocular tumours.

There are two major limitations to our study. First, despite being daily confirmed by newscasts and by other means of communication, the analyses we did are based on a single-centre experience, thus the results may not reflect the overall Italian ophthalmologic quality of care. Second, our data lacked information on the severity of the ophthalmic conditions the patients suffered from. Moreover, in this study, we did not display any data regarding the effects of the lockdown measures on two of our major surgical services (i.e. vitreoretinal and glaucoma surgery). In fact, this kind of analysis was performed by another recent paper we contributed to [6]. In the latter, the authors reported a significant reduction in surgical activity during the pandemic.

In conclusion, a significant number of patients could not get adequate ophthalmic treatment during the Lockdown Period. This might lead to a scenario where we will have to face an increasing amount of severe ocular diseases in the next future. Thus, some questions come to mind: Will patients with chronic health conditions actually have more severe consequences from their disease? What will the financial impact on public health providers be? Will telemedicine live up to this scenario? These questions open a new perspective on the management of ocular and non-ocular disorders in the era of the COVID-19 Pandemic.

## Data Availability

The data that support the findings of this study are openly available in Mendeley Data and can be retrieved from http://dx.doi.org/10.17632/wmys4zc68n.1.
